# Pediatric functional magnetic resonance neuroimaging: tactics for encouraging task compliance

**DOI:** 10.1186/1744-9081-7-10

**Published:** 2011-05-06

**Authors:** Michael W Schlund, Michael F Cataldo, Greg J Siegle, Cecile D Ladouceur, Jennifer S Silk, Erika E Forbes, Ashley McFarland, Satish Iyengar, Ronald E Dahl, Neal D Ryan

**Affiliations:** 1 Department of Psychiatry, University of Pittsburgh School of Medicine, Pittsburgh PA, USA; 2 Department of Behavioral Psychology, Kennedy Krieger Institute, Baltimore MD, USA; 3 Department of Psychiatry, Johns Hopkins School of Medicine, Baltimore MD, USA; 4 Department of Behavior Analysis, University of North Texas, Denton TX, USA; 5 Department of Psychology, University of Pittsburgh, Pittsburgh PA, USA; 6 Department of Statistics, University of Pittsburgh, Pittsburgh PA, USA

## Abstract

**Background:**

Neuroimaging technology has afforded advances in our understanding of normal and pathological brain function and development in children and adolescents. However, noncompliance involving the inability to remain in the magnetic resonance imaging (MRI) scanner to complete tasks is one common and significant problem. Task noncompliance is an especially significant problem in pediatric functional magnetic resonance imaging (fMRI) research because increases in noncompliance produces a greater risk that a study sample will not be representative of the study population.

**Method:**

In this preliminary investigation, we describe the development and application of an approach for increasing the number of fMRI tasks children complete during neuroimaging. Twenty-eight healthy children ages 9-13 years participated. Generalization of the approach was examined in additional fMRI and event-related potential investigations with children at risk for depression, children with anxiety and children with depression (N = 120). Essential features of the approach include a preference assessment for identifying multiple individualized rewards, increasing reinforcement rates during imaging by pairing tasks with chosen rewards and presenting a visual 'road map' listing tasks, rewards and current progress.

**Results:**

Our results showing a higher percentage of fMRI task completion by healthy children provides proof of concept data for the recommended tactics. Additional support was provided by results showing our approach generalized to several additional fMRI and event-related potential investigations and clinical populations.

**Discussion:**

We proposed that some forms of task noncompliance may emerge from less than optimal reward protocols. While our findings may not directly support the effectiveness of the multiple reward compliance protocol, increased attention to how rewards are selected and delivered may aid cooperation with completing fMRI tasks

**Conclusion:**

The proposed approach contributes to the pediatric neuroimaging literature by providing a useful way to conceptualize and measure task noncompliance and by providing simple cost effective tactics for improving the effectiveness of common reward-based protocols.

## Background

Functional magnetic resonance imaging is increasingly being used to advance our understanding of normal and pathological brain function and development in children and adolescents. However, there are a number of challenges that clinicians and researchers encounter. Noncompliance involving an inability to remain in a scanner to complete fMRI tasks is one common and significant problem. This paper describes the development and application of an approach we believe may improve the effectiveness of conventional reward-based approaches used to encourage task compliance. We discuss some issues surrounding task noncompliance and offer that integrating tactics derived from learning based behavior therapies into conventional reward-based protocols may help encourage compliance. Using a case study design, preliminary results show improvements in the number of study tasks completed in healthy children and those with clinical disorders, providing proof of concept data for the recommended tactics. While the present discussion focuses primarily on task compliance during functional neuroimaging investigations, some of the problems discussed and recommendations may generalize to other forms of neuroimaging (e.g., clinical MRI, PET, DTI).

### Task noncompliance in pediatric functional neuroimaging

Noncompliance has been recognized as a central issue within pediatric fMRI research [1-5], as well as clinical MRI procedures [6], for some time. It extends from difficulties entering a scanner to completing fMRI tasks, performing tasks accurately and remaining motionless. Many interventions have been developed to enhance cooperation. These include cognitive-behavior modifications such as relaxation [7] as well as play therapy [8], observing a role model [9] and providing scanner exposure/simulation [10-12]. Reinforcement based protocols are commonly used to help children learn to minimize head motion [13-16]. There are also comprehensive packages that bring together many different basic techniques, providing for a more systematic approach [e.g., [17]]. One important gap, however, in the pediatric imaging literature concerns why task noncompliance occurs and what ways are available to intervene. While many recognize task noncompliance as a problem, it has only received a cursory treatment in the pediatric neuroimaging literature. This is rather surprising and unfortunate given that increases in task noncompliance produce a greater risk that a study sample is not representative of the study population.

Our understanding of the prevalence of task noncompliance within the pediatric neuroimaging literature is another area of weakness. Several functional neuroimaging studies have reported that task compliance improves with age and is higher in typically developing children relative to variety of clinical populations [3,5,14]. While this seems reasonable, the picture remains somewhat clouded because definitions of task compliance vary [18]. For example, one investigation [9] defined compliance 'success' as completing an anatomical scan and at least one or more of four total scheduled tasks. By comparison, another investigation [18] defined compliance as completing a whole battery of fMRI tasks that produced interpretable data for inclusion in group statistical analyses. These differences in definition have several potential negative consequences for pediatric functional neuroimaging research. The first is that reported success rates for a particular age group or clinical population may vary markedly across investigations. The second is it prevents meaningful evaluation of any intervention for noncompliance and complicates comparisons between interventions.

### Conventional reward-based protocols in pediatric neuroimaging

In all pediatric functional neuroimaging studies, researchers and clinicians use rewards to encourage and maintain task compliance. This highlights an important tie to reinforcement learning theories. The widespread application of reward protocols also highlights recognition of the relationship between task compliance and a sufficiently rewarding neuroimaging environment. Accordingly, conventional reward-based protocols often employ multiple sources and different kinds of rewards to encourage motivation and task completion. One source is the monetary compensation provided for participation [e.g., [19]]. Task compliance may also be influenced with monetary rewards earned directly as a result of performance on an fMRI task [e.g., [20]]. Researcher-identified rewards (stickers, glow-pens, gift certificates, coloring books or brain pictures) represent yet another major source of reward [e.g., [17]]. Lastly, there are social rewards, which include words of encouragement and verbal praise for working hard, good performance and remaining in the scanner.

Reward-based approaches clearly help to create a positive, encouraging, and supportive environment necessary for successful pediatric neuroimaging research. It seems important to note, however, that for a significant number of children, especially young children and sensitive clinical populations, reward-based approaches will not be enough to promote task compliance. Nevertheless, there still may be ways of improving or strengthening existing conventional reward-based approaches. Results of developmental studies on reinforcement processes, tactics used in behavior therapy for children and head motion training preparations that tap reinforcement as a change agent offer some important insights into why a reward-based protocol may fail and how to improve its effectiveness.

One of the ways a researcher-identified reward, such as sticker or trinket, may fail to maintain task compliance is that the reward does not have the capability to function as a reinforcer, which strengthens or makes a behavior more likely to occur (e.g., completing an fMRI task). Thus, while a subject may report that they 'like' or 'want' a preselected reward, it may simply not encourage or maintain a target behavior. In fact, finding appropriate rewards that work as reinforcers is a major component of effective sticker charts [21-23]. A second reason why a researcher-identified reward may fail is that the subject may view the reward as desirable or valuable 'now,' but because it is not earned until 'later,' the subjective value of the reward may plummet over time, along with its potentially reinforcing function. Loss of value over time is referred to as temporal discounting and evidence from developmental studies has shown that delayed rewards are discounted to a greater extent in young children (6-11 years) as compared with adolescents (12-17 years) [24]. This may partly account for reported reductions in task compliance in younger children. A third related reason why the value of a researcher-identified reward may diminish is that subjects encounter mounting demands associated with participation, such as a remaining motionless, remaining in the scanner for a long period and completing multiple, often effortful (and boring) tasks. With these cumulative demands/costs, reward value may diminish and performance breaks down. Recent evidence from a large scale developmental study (N = 849; 4-14 years) shows motivational differences to monetary reward in children as a function of age and gender, with older children and males more resistant to higher response costs [25]. Finally, competition among rewards can also influence task compliance. Participation in a research study is essentially the choice of one rewarding activity over another. Noncompliance can emerge when study-rewards cannot compete with more valued concurrent non-study rewards, such as visiting friends, or delayed non-study rewards, such as going to dinner after the study.

In this paper, we describe how integrating tactics derived from learning based behavior therapies into conventional reward-based protocols may help to improve or maintain task compliance. These tactics include (1) using preference assessments to identify multiple subject-specific rewards, (2) increasing reinforcement rates during imaging by providing a reward for each task, and (3) presenting a visual 'road map' during imaging that lists tasks, associated rewards and progress. For brevity, this collection of tactics will be referred to as the multiple reward compliance protocol. In what follows, we describe the development and application of our approach in several groups of children that participated in an fMRI investigation. Our results showing increases in the percentage of fMRI tasks completed in several groups of children provide proof of concept data for the multiple reward compliance protocol. Additional support is provided by results showing our approach generalized to several additional fMRI and event-related potential investigations and clinical populations (children at risk for depression, children with anxiety and children with depression).

## Methods

### Subjects

Twenty-eight healthy children ages 9-13 years (mean 11.1, SD = 1.83) participated. Participation required completing a battery of clinical assessments and a 2 hr 3T functional neuroimaging session. Exclusion criteria for the study included: (a) symptoms suggestive of an Axis I psychiatric disorder based on parent report on the Child or Adolescent Symptom Inventory-4 [26,27], (b) the existence of a major systemic medical illness, (c) a history of serious head injury, (d) having eye problems or difficulties in vision not corrected by the use of glasses or contact lenses, measured as vision of 30/20 or better with both eyes open using a hand-held eye-chart or (e) metal or devices contraindicated for MRI. All participants were recruited from community advertisements. After a detailed description of the study and before participation, parents gave written informed consent for their child's participation in the study. Children gave written informed assent. All studies reported were approved by the University of Pittsburgh Institutional Review Board.

### Primary study groups

The twenty-eight subjects recruited for the study were subdivided into three groups based upon their order of recruitment. Table 1 provides demographic information for each group. Groups were constructed based upon changes made in the reward protocol and fMRI tasks to facilitate remaining in the scanner to complete tasks. Compliance in the first twelve subjects and, two later additional subjects, was encouraged using a conventional reward-based protocol--see below. This is the "Reward" protocol group (N = 14). A second group also received the conventional reward-based protocol and the multiple reward compliance protocol (MRCP)---see below. This group was designated the "MRCP" group (N = 5). Finally, the third group was designated the "MRCP Plus" group (N = 9). This latter group received the conventional reward-based protocol, the MRCP and three of the seven fMRI tasks were shortened in duration. This action decreased the total time needed to complete all fMRI tasks from 66.7 min to 55.9 min, working under the idea that reducing task demand may enhance task compliance.

**Table 1:** Demographics and compliance results

Grouping	Groups		N	Age Range	Mean Age (SD)	Total Duration	Tasks	Average Compliance*
Primary Group	Reward group		14	9-13 yrs.	11.1 (1.78)	120 min.	7	68%
	MRCP group		5	9-13 yrs.	11.3 (2.15)	120 min.	7	97%
	MRCP Plus group		9	9-13 yrs.	11.2 (1.55)	120 min.	7	94%
	MRCP Combined group ^		14	9-13 yrs.	11.2 (1.54)	120 min.	7	95%
Generalization Groups+	Depression Study 1 group		34	10-15 yrs.	12.5 (1.91)	90 min.	4	100%
	Depression Study 2 group		32	9-17 yrs.	14.1 (1.98)	90 min.	5	100%
	Anxiety Study:	fMRI group 1	21	9-13 yrs.	10.8 (1.32)	120 min.	6	82%
		fMRI group 2	33	9-13 yrs.	10.3 (1.30)	120 min.	6	86%
		ERP	54	9-13 yrs.	10.5 (1.31)	150 min.	3	100%

^ Pooling of MRCP and MRCP Plus groups

*Total number of fMRI or ERP tasks completed/total number of tasks

+All groups received a version of the MRCP

### Generalization test groups

Table 1 also provides demographic information and percent compliance data for several additional groups of children from three different investigations. The first two groups participated in two separate fMRI studies on child and adolescent depression. Both studies involved completing four or five tasks during a 90 minute 3T fMRI session. The first group included 34 subjects ages 10-15 years with half at high familial risk for depression. The second group included 32 depressed and non-depressed subjects ages 9-17 years. Generalization was further tested in a large scale NIMH funded investigation on childhood anxiety. Fifty-four children ages 9-13 years diagnosed with anxiety completed (a) a 2 hour 3T fMRI session that required completing six tasks, and (b) a 2.5 hour ERP session that required completing three tasks.

### Tasks and compliance measure

For the primary study groups, seven tasks were presented during neuroimaging in a randomized order, each separated by rest periods that lasted up to 5 minutes. The tasks used assessed attention, reward sensitivity, threat processing and discriminated avoidance and approach [28]. Compliance was defined as remaining in the scanner during the time period a particular task was presented. Each task completed was considered one "success"----regardless of whether the task was spread over multiple neuroimaging runs. Percent compliance was calculated by dividing the number of tasks completed successfully (N of successes) by the total number of programmed tasks (our N = 7; total possible successes). Defining task compliance in this way sets task compliance apart from problems related to excessive head movement and performance accuracy---each of which are distinguishable by their own dependent measures. This approach also differs from other approaches which include head motion and task performance in defining success [18].

### Conventional reward-based protocol

The general approach we employed to encourage and maintain compliance followed many conventional practices [1-5,17] with most subjects receiving simulator training (i.e., being placed in a mock scanner and hearing scanner noises for approximately 5 min) and all receiving pretraining on select imaging tasks. Subjects also received as part of the reward-based protocol monetary compensation for participation, a picture of their brain and verbal support and encouragement for working hard, good performance and remaining in the scanner.

### Multiple reward compliance protocol

As noted above, conventional reward-based protocols can fail to maintain task compliance for a variety of reasons---researcher-selected rewards may be inappropriate, discounted, devalued or uncompetitive. Fortunately, basic and clinical research studies on reinforcement processes and tactics used in learning based behavior therapies for children suggest some tactics for enhancing conventional reward-based protocols. Below we describe the rationale behind the development of a multiple reward compliance protocol. Critical elements of the protocol include (1) using preference assessments prior to imaging to identify multiple subject-specific rewards, (2) increasing reinforcement rates during imaging by providing a reward for each task, and (3) presenting a visual 'road map' during imaging that lists tasks, associated rewards and current progress.

#### Identifying preferred rewards

One approach to adverting task noncompliance issues related to selection of an ineffective reward(s) is to let subjects identify their own. Clinicians developing behavioral treatments for typically developing children and those with cognitive dysfunction commonly employ 'preference assessments' to identify preferred stimuli that may serve as subsequent reinforcers [29]. Accordingly, the first element of the multiple reward compliance protocol involved identifying multiple subject-preferred rewards. The preference assessment implemented prior to neuroimaging required subjects to select seven preferred toys (one for each fMRI task run) from a large drawer containing small toys, such as stickers, rubber balls, glowing pens...(items costing ~$1.00). This approach effectively eliminated any guesswork about reward value by using choice as an index of subjective value. Incidentally, the order of reward selection provides insight into which items are viewed more favorably, potentially highlighting those rewards with greater motivational properties. Following selection, a preference hierarchy can be constructed by requiring children to physically order rewards from most to least preferred. The resulting preference hierarchy may prove especially useful under conditions where some neuroimaging tasks are markedly more difficult than others and allow the pairing of more difficult tasks with most preferred selections to enhance motivation.

#### Reducing reward delays and increasing reinforcement rate

One approach to combating decreases in reward effectiveness caused by delays or cost/effort is by reducing delays and increasing the number of rewards earned per unit time. While increasing the frequency of verbal praise, encouragement and feedback can be effective in facilitating task compliance, a potentially more effective approach is to use preference assessment results to pair selected rewards with fMRI tasks. Accordingly, the second element of the multiple reward compliance protocol involved pairing subject-selected rewards highlighted in the preference assessment with imaging tasks and informing subjects they will earn one of their chosen rewards after completing each task. Providing multiple rewards during imaging effectively insulates rewards against the negative effects of temporal delays and cost/effort by increasing the local rate of reinforcement (i.e., number of rewards earned per minute during a session). In effect, the multiple reward compliance protocol approach models "catch them being good" clinical approaches that stress providing high rates of reinforcement for appropriate behavior [21-23].

#### Visual progress display

**Figure 1:**
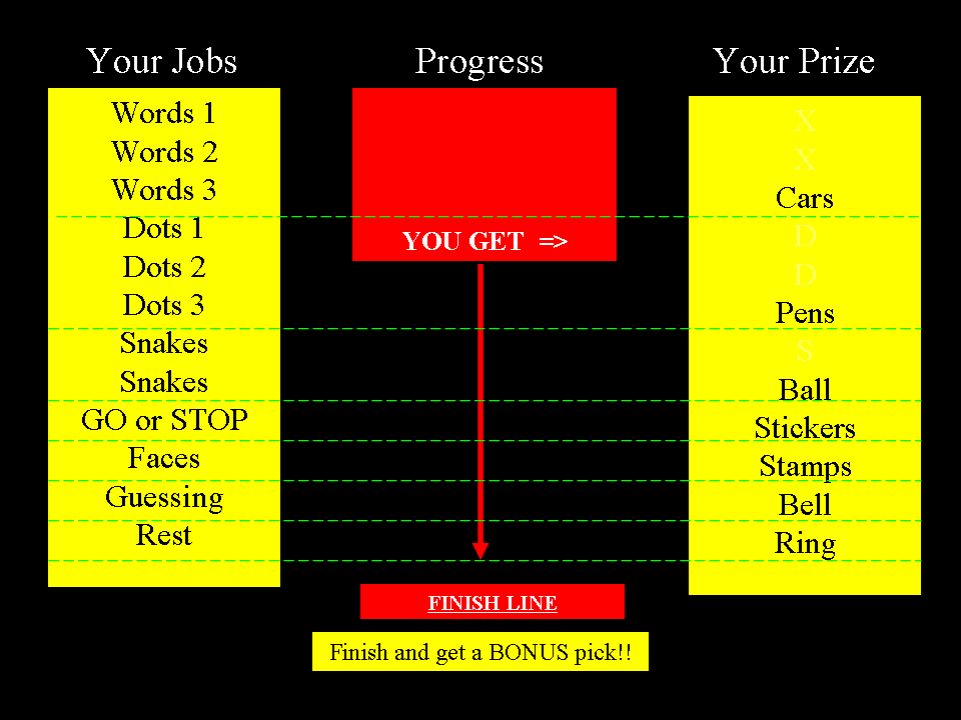
**Visual progress display presented during neuroimaging rest periods**. Informational display highlighting each experimental task, a progress bar and subject-identified rewards earned for completing tasks. The display was presented between imaging tasks and functioned as a supplement to researcher's instructions and verbal praise/encouragement for participation. Tasks are listed by imaging runs, such that those requiring multiple runs are listed multiple times. Task-paired rewards, listed as 'prizes,' were identified prior to neuroimaging using a preference assessment procedure and were earned contingent upon task completion, regardless of performance accuracy. The progress bar descended after a task was completed.

Considerable developmental research shows age-related improvements in memory and information processing speed and reductions in susceptibility to interference [30,31]. Within any neuroimaging investigation, children are exposed to a wealth of information and demands that can be overwhelming and aversive, prompting noncompliance. Researchers routinely manage task information and demands using verbal communication during imaging rest periods, informing subjects about current and upcoming tasks as well as providing reassurance about progress (e.g., "You finished that task. Just three more tasks left. You are doing great!"). Accordingly, the third element of the multiple reward compliance protocol involves supplementing verbal communications with a visual presentation that highlights information about tasks, demands and progress. Figure 1 provides an illustration of a visual progress display we presented to subjects during imaging rest periods, which can be easily created and presented using Microsoft Word^®^, Powerpoint^® ^or programmed using Eprime^®^. The format presents information about the pairing of tasks with subject-identified rewards and current progress. Each task is listed (under 'Jobs') and separated by imaging run, such that tasks requiring multiple imaging runs are listed multiple times. In the center of the display is a progress bar the researcher can advance downward toward a 'finish line' after each task is completed. Presentation of the visual display during breaks enables subjects to quickly observe their progress, tasks, earned rewards and upcoming rewards. The information provided about upcoming tasks may also insulate against the development of negative affective responses associated with the uncertainty of future events and provide an increased sense of control. At least one published protocol has employed a version of this approach with a "virtual sticker chart" that highlights progress [17]. One strength behind the sticker chart approach is the progress information it provides. However, a potentially important weakness is that stickers provided may lack rewarding or reinforcing properties for some subjects.

## Results

### Application of the multiple reward compliance protocol

**Figure 2:**
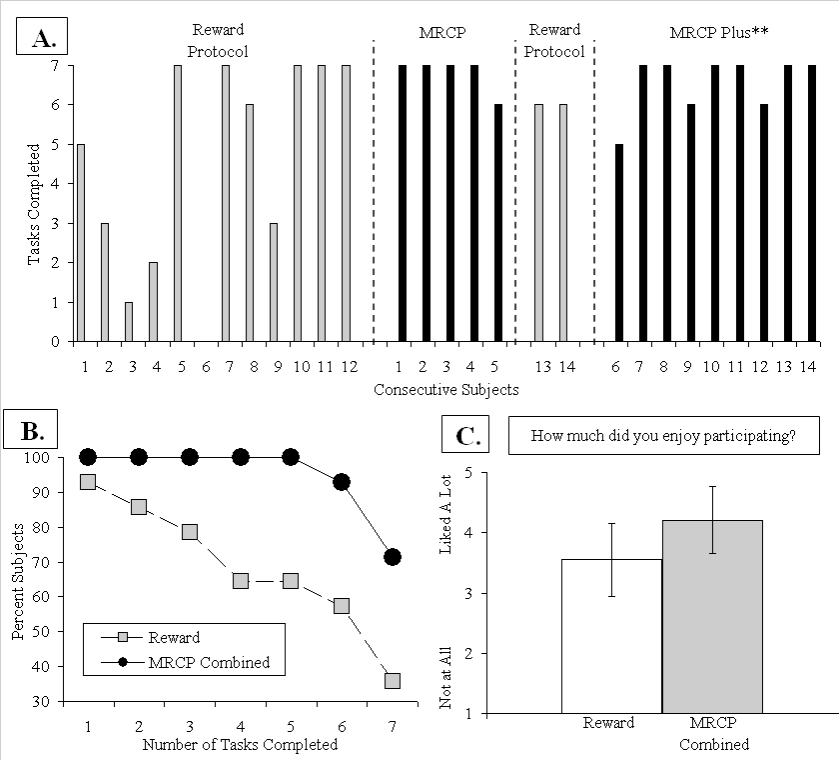
**Illustration of improvements in compliance in youths (9-13 years old)**. Panel A provides a time line of the number of fMRI tasks completed by consecutive subjects. Subjects in the Reward group received a conventional reward-based protocol to encourage compliance that consisted of monetary compensation for participation, customary verbal feedback praise/encouragement, simulator training and exposure to select tasks prior to imaging. Subjects in the *multiple reward compliance protocol *or MRCP group also received the reward-based protocol along with earning a subject-selected reward following completion of each fMRI task and presentation of a visual progress display (see Figure 1). The duration of three tasks was later shortened in the MRCP Plus** subjects (6-14), which decreased total time of tasks from 66.7 min to 55.9 min. Panel B shows significant differences in tasks completed between Reward and the MRCP Combined group (pooled MRCP and MRCP Plus groups). Panel C shows a slightly more favorable, but not significant, view of the experiment by the MRCP Combined group. Bars reflect standard deviations.

Table 1 provides demographic information and information on percent task compliance for each group. In general, results show a higher percentage of fMRI tasks were completed in groups that received the multiple reward compliance protocol, providing some proof of concept data for the approach. Panel A in Figure 2 shows task compliance data for consecutive subjects. Percent task compliance in the Reward group averaged 68.4% (SD = 35.5%), the MRCP group averaged 97.1% (SD = 6.4%) and the MRCP Plus group averaged 93.6% (SD = 10.4%). Panels B and C in Figure 2 provide an additional perspective on our efforts to improve and maintain compliance using the MRCP. Panel B in Figure 2 shows survival curves for the Reward group and pooled data from the MRCP group and the MRCP Plus group (hereafter termed the 'MRCP combined' group). The rationale for pooling groups for this analysis was to match the group size of  the Reward group (N = 14), which seems appropriate given that both groups received the MRCP whereas the Reward group did not. Results of the analysis provide a rudimentary picture of success when faced with multiple tasks. Plotted is the percentage of subjects in each group that completed one to all seven tasks during neuroimaging. The function reveals 90% of Reward group subjects initially completed 1-2 tasks, but as the number of tasks increased the percentage dropped to 35%. By comparison, the MRCP combined group consistently completed more tasks overall.

To explore the level of compliance improvement, we used a censored geometric distribution model to estimate the probability of task compliance. The analysis revealed that 11.8% fewer participants were estimated as likely to complete each consecutive task in the Reward group compared to only 4.1% for the MRCP combined group. Bootstrap analysis (10,000 iterations) suggested the percentage of subjects that end participation is significantly higher in Reward group than the MRCP combined group (*p *< 0.05), and the difference of percentage has a 95% CI of (1.1%, 15.9%). As a result, about 41.52% of subjects would be predicted to finish all seven tasks in the Reward group, while 74.6% of subjects would be predicted to finish all seven tasks in the MRCP combined group. Lastly, panel C in Figure 2 shows ratings assessing how 'enjoyable' participation was on a four point post-experimental questionnaire (1 = Not at all, 3 = No Feelings, 5 = A Lot). Ratings for subjects in the MRCP combined group (N = 14; M = 4.2, SD = 1.05) were more favorable, but not significantly (*p *> 0.05), than ratings for subjects in the Reward group (N = 12, two cases missing; M = 3.5, SD = 1.24).

### Generalization tests of the multiple reward compliance protocol

While we did not use a randomized controlled trial, improvements in the percentage of fMRI tasks completed across subjects suggests the multiple reward compliance protocol may have some utility for encouraging children to complete tasks. The relative ease and low cost of employing the multiple reward compliance protocol suggests its major utility may be as a supplemental 'insurance' policy to existing methods. One of ways to evaluate its utility is to determine whether the findings above represent an isolated case, resulting from some aspect of our procedure, a research assistant or some feature of the imaging environment. The multiple reward compliance protocol may have limited value if it cannot be shown to generalize to other investigations that employ different research personnel, different types of neurophysiological techniques and more challenging pediatric populations, such as children with anxiety or depression. Results highlighting generalization of the multiple reward compliance protocol would provide additional proof of concept data for the approach.

To examine this issue, the multiple reward compliance protocol was applied in a number of additional fMRI and event-related potential (ERP) investigations that employed different research assistants and pediatric populations (e.g., children at risk for depression, children with depression and children with anxiety). For all studies, task compliance was again defined as the percentage of total scheduled tasks completed. Table 1 provides demographic information and percent compliance data for each study. The first two fMRI studies focused on child and adolescent depression. In these studies, the multiple reward compliance protocol was administered without the visual display component. Both studies involved completing four or five tasks during a 90 minute fMRI session. Results presented in Table 1 show task compliance was 100%. Generalization was further tested in a large scale NIMH funded investigation on childhood anxiety. Fifty-four children with anxiety completed (a) a 2 hour fMRI session that required completing six tasks, and (b) a 2.5 hour ERP session that required completing three tasks. Results presented in Table 1 show the first 21 consecutive subjects that underwent fMRI, with some portion not receiving the visual display due to a procedural error, averaged 82% (SD = 36%) task compliance. Within this group, 17 of the 21 subjects (81%) completed five or six tasks. Results presented in Table 1 show the next group of 33 consecutive subjects that underwent fMRI averaged 86% (SD = 31%) task compliance. Within this group, 27 of the 33 subjects (82%) completed five or six tasks. Finally, task compliance with all three ERP tasks was 100% for all 54 subjects. Overall, the consistently high level of task compliance attained across three different investigations, two different methodologies (fMRI, ERP) and four different pediatric populations (healthy children, children at risk for depression, children with anxiety and children with depression) offers some preliminary support for the multiple reward compliance protocol.

## Discussion

Noncompliance is a common phenomenon in pediatric neuroimaging [1,2]. Recognition of the problem has prompted many discussions on structuring a positive and supportive environment for children [1-5,17] as well as generated specific tactics, such as those for reducing excessive head motion [9-12]. In this paper, we focused on task noncompliance, defined as the inability to remain in a scanner to complete fMRI tasks. It is an especially significant problem in pediatric functional magnetic resonance imaging research because increases in task noncompliance produces a greater risk that a study sample will not be representative of the study population. Consequently, efforts to develop procedures for improving task noncompliance address an important gap in the pediatric neuroimaging literature. The approach we presented provides some heuristics for understanding noncompliance and demonstrated tactics for addressing tack noncompliance in pediatric neuroimaging.

One contribution of our approach to pediatric neuroimaging was our focus on task noncompliance as an issue worth addressing apart from other forms noncompliance. Within the pediatric imaging literature, the term noncompliance encompasses a wide range of problems encountered by researchers and clinicians. These may include failures to enter a scanner, remain in the scanner, complete tasks, follow task instructions accurately and remain motionless. While all forms of noncompliance are significant barriers, it seems reasonably well established that maintaining participation involves addressing many problem areas with different types of tactics and doing so at different times. For example, some approaches encourage cooperation early in the process by having subjects watch a video of a child completing a routine fMRI study in a scanner [5]. Another recommended tactic to promote adherence during fMRI involves presenting a virtual sticker chart during rest periods to highlight current progress [17]. Just as these examples focused on a specific aspect of participation, we focused on encouraging subjects to complete fMRI tasks---measured as the percentage of total fMRI tasks completed---by using more preferred and more task-specific rewards. An important shared feature of these approaches and our multiple reward compliance protocol is that each is not designed to address all forms of noncompliance, which would include task performance and head motion. Such an expectation is too demanding. By targeting only one form of [potential] noncompliance for improvement, what these approaches and our multiple reward compliance protocol may lose in generality, meaning their ability to remediate poor task performance and excessive head motion, they gain in effectiveness.

Another contribution of our approach is that it highlights the idea that task noncompliance may result from less optimally designed reward-based protocols. This approach emphasizes the importance of creating an environment conducive to completing study tasks which differs in focus from other perspectives that might emphasize subject-related factors such as fear, anxiety or boredom as contributors to task noncompliance. In our case, task compliance was viewed as emerging from an imaging environment that provides adequate and sufficient reward. The end result was an enhanced reward protocol that included (1) a preference assessment to identify multiple subject-specific rewards, (2) increasing reinforcement rates during imaging by providing a reward for each task, and (3) presenting a visual 'road map' during imaging that lists tasks, associated rewards and progress. It is equally important to recognize that while our findings may not directly support the effectiveness of the multiple reward compliance protocol, our rationale is supported by the behavior therapy literature and basic behavioral research on learning processes. All of this is not to say that fear or anxiety are irrelevant to our understanding of noncompliance or that our tactics should replace using relaxation techniques or exposure training. It is merely to point out that improving reward protocols can contribute to enhancing task compliance.

## Limitations

There were several limitations of the present investigation that restrict the conclusions that can be drawn about the multiple reward compliance protocol. The principal limitation was our inability to use a randomized control trial during the course of our investigation, which leaves results open to biases and experimenter expectancies normally controlled for with randomized control trial. Nevertheless, we submit that our results showing a higher percentage of fMRI task completion by healthy children provides proof of concept data for the recommended tactics. Additional support was also provided by results showing our approach generalized to several additional fMRI and event-related potential investigations and clinical populations (children at risk for depression, children with anxiety and children with depression). This level of generalization suggests that the multiple reward compliance protocol may extend to populations with significant cognitive impairments, such as children with developmental disabilities who are at increased risk of hypoxia when sedation approaches are used [32]. Another limitation of the present investigation concerns the relative contributions of the various components of the multiple reward compliance protocol (preference assessment, increase in reward, visual display). Future research is needed that examines which component or components may contribute the most to improving task compliance. During our generalization tests, there was a rather substantial sample of subjects that did not receive the visual display component but nonetheless showed high task compliance. This was limited to the two fMRI studies on child and adolescent depression (N = 34 and N = 32) and a portion of the first group of children participating in the childhood anxiety study (N = 21). Findings showing high task compliance without the visual display component suggest identifying and providing rewards contingent upon task completion may be relatively more important than the information supplied by the road map. Another limitation of the present investigation is that it remains unclear what subject variables or aspects of participation contributed to task noncompliance. Researchers and clinicians might benefit from gathering information during debriefing about the reason(s) for early termination. Children's reports may contain valuable information about how to structure the environment or tasks in ways that encourage cooperation.

## Conclusions

Functional magnetic resonance imaging is increasingly being used to advance our understanding of normal and pathological brain function and development in children and adolescents. Noncompliance involving an inability to remain in a scanner to complete fMRI tasks is one common and significant problem. Consequently, researchers and clinicians devote considerable effort to developing a supportive, positive environment. We proposed that some forms of task noncompliance may emerge from less than optimal reward protocols. Our findings suggest that increasing our attention to how rewards are selected and delivered may aid cooperation with completing fMRI tasks. The approach presented and preliminary findings contribute to the pediatric neuroimaging literature by providing a useful way to conceptualize and measure task noncompliance and a set of cost effective tactics for improving the effectiveness of common reward-based protocols.

## List of abbreviations used

MRCP: Multiple reward compliance protocol

## Competing interests

The authors declare that they have no competing interests.

## Authors' contributions

Design: MS, MC, GS. Data collection: MS, AM, JS, CL, GS. Analysis: MS, GS, IS.

Writing: MS, GS, MC, CL, JS, EF, RD and NR All authors read and approved the final manuscript.
